# Valuing Diversity and Inclusion in Health Care to Equip the Workforce: Survey Study and Pathway Analysis

**DOI:** 10.2196/34808

**Published:** 2022-05-06

**Authors:** Jiban Khuntia, Xue Ning, Wayne Cascio, Rulon Stacey

**Affiliations:** 1 Health Administration Research Consortium Business School University of Colorado Denver, CO United States; 2 Business Department University of Wisconsin Parkside Kenosha, WI United States

**Keywords:** health system, workforce, workplace, diversity, inclusion, improve, recruit, collaborate, health care, worker, employee, CEO, chief executive officer, United States, North America, characteristic, benefit, influence, strategy, pathway, hiring, hire, collaboration, talent, student

## Abstract

**Background:**

The COVID-19 pandemic, with all its virus variants, remains a serious situation. Health systems across the United States are trying their best to respond. On average, the health care workforce is relatively homogenous, even though it cares for a highly diverse array of patients. This perennial problem in the US health care workforce has only been accentuated during the COVID-19 pandemic. Medical workers should reflect on the variety of patients they care for and strive to understand their mindsets within the larger contexts of culture, gender, sexual orientation, religious beliefs, and socioeconomic realities. Along with talent and skills, diversity and inclusion (D&I) are essential for maintaining a workforce that can treat the myriad needs and populations that health systems serve. Developing hiring strategies that will help achieve greater workforce diversity remains a challenge for health system leaders.

**Objective:**

The primary aims of this study were to: (1) explore the characteristics of US health systems and their associations with D&I practices and benefits, (2) examine the associations between D&I practices and three pathways to equip workforces, and (3) examine the associations between the three pathways to better equip workforces and business and service benefits. The three pathways are: (1) improving D&I among existing employees (IMPROVE), (2) using multiple channels to find and recruit the workforce (RECRUIT), and (3) collaborating with universities to find new talent and establish plans to train students (COLLABORATE).

**Methods:**

During February to March 2021, 625 health systems in the United States were surveyed with the help of a consultant, 135 (21.6%) of whom responded. We assessed workforce talent- and diversity-relevant factors. We collected secondary data from the Agency for Healthcare Research and Quality Compendium of the US Health Systems, leading to a matched data set of 124 health systems for analysis. We first explored differences in diversity practices and benefits across the health systems. We then examined the relationships among diversity practices, pathways, and benefits.

**Results:**

Health system characteristics such as size, location, ownership, teaching, and revenue have varying associations with diversity practices and outcomes. D&I and talent strategies exhibited different associations with the three workforce pathways. Regarding the mediating effects, the IMPROVE pathway seems to be more effective than the RECRUIT and COLLABORATE pathways, enabling the diversity strategy to prompt business or service benefits. Moreover, these pathway effects go hand-in-hand with a talent strategy, indicating that both talent and diversity strategies need to be aligned to achieve the best results for a health system.

**Conclusions:**

Diversity and talent plans can be aligned to realize multiple desired benefits for health systems. However, a one-size-fits-all approach is not a viable strategy for improving D&I. Health systems need to follow a multipronged approach based on their characteristics. To get D&I right, proactive plans and genuine efforts are essential.

## Introduction

### Background

Health systems have been overwhelmed with COVID-19 patients [1]. Perennial shortages in the health care workforce have been exacerbated during the pandemic [2]. Stress, trauma, and burnout have tested the limits of health systems’ existing workforces [3], and health systems lack workforces to treat the diversity of COVID-19 patients [4,5].

In general, the workforce in medicine is relatively homogenous, despite serving diverse populations. The health care system faces significant challenges matching patients’ beliefs, attitudes, expectations, and care customization to an appropriately diverse workforce. In 2020, the US health care workforce reportedly comprised more than 50% white, approximately 20% Asian, 7% Black, and less than 1% Hispanic and Native American workers [6]. Two-thirds of physicians and surgeons are Christian, 14% are Jewish, and fewer than 15% represent other religions [7]. In addition, two-thirds are men, although this is changing as more women are admitted to medical schools [8]. In addition, dropouts among medical students in the first 2 years are high due to socioeconomic factors [9]. Assessment of sexual and gender diversity is also problematic, as disclosures risk discrimination claims [10], although schools attract unrepresented LGBTQ applicants [11]. In general, a lack of diversity in the health care workforce poses challenges for caring for diverse populations of patients, leading to variable and often detrimental access and quality issues [12]. Although the value of diversity has been well-established, unless health system leaders adopt explicit strategies to improve diversity and inclusion (D&I), they will not accomplish this goal. Moreover, it is not clear how health systems can best equip their workforces along with best practices to achieve a diverse workforce.

This study sought to assess efforts to improve D&I, as reported by chief executive officers (CEOs) of health systems across the United States. We argue that in addition to the talent and skills required for effective health care delivery, D&I needs to be part of the strategic agenda. Without this consideration, catering to the diverse needs of various populations will continue to present a challenge. This study thus explored the characteristics of US health systems and the perceived benefits of D&I. To achieve a diverse workforce in health care, health systems need to leverage different pathways. We examined factors that may shape those pathways to help balance talent and diversity. We also explored the associations between workforce pathways and both business and service benefits. Our approach will provide decision-makers with helpful practice and policy inputs [12,13].

### Health Care Workforce Diversity

Health disparities are not homogeneous. Segments of populations are affected differently by different diseases. Accordingly, approaches and treatments may vary across these segments and thus require customized care [13]. Therefore, it stands to reason that a lack of diversity in the health care system can negatively affect patients. For instance, an Indian patient with traditional ethnic or religious values or a transgender patient may have needs unique to their circumstances and worldview. A diverse workforce in health systems should respectfully and knowledgeably approach and assist all patients with an appreciation of their values and needs [12]. Professionals from different cultures and backgrounds bring unique perspectives to share with colleagues and patients alike as they strive to better understand and respond to patients’ needs.

Alarmingly, when patients do not find providers, approaches, or treatments that echo or align with their beliefs, culture, or life circumstances, they are more prone to delay or avoid care. This problem is inherent in the current health care system. Patients from different cultures may perceive diseases and treatments differently. Greater diversity among health care workers will help reduce the barriers patients face when seeking care and contribute to better access and quality of care.

Prior research suggests that health care workforce diversity can improve creativity and decision-making while catering to multiple perspectives and contexts [14,15]. Specific to the COVID-19 context, research suggests that diversity-oriented leadership could improve employees’ knowledge-sharing, promote professional collaborations, and help reach marginalized and hard-to-reach communities [16,17]. For example, immigrant and refugee professionals represent essential resources that can provide linguistic and cultural services for their communities during and after the COVID-19 pandemic [18]. Greater diversity broadens traditional boundaries to improve care and patient satisfaction, and could prove helpful in managing stressful environments [4,5].

Employee engagement is also higher in organizations with diverse workforces [19]. As the populations served by doctors are becoming increasingly diverse, doctors need to adopt a more global mindset. Ensuring a diverse student body in medical schools will help future doctors broaden their perspectives and improve their understanding of D&I. Doctors from such schools will be better equipped to provide care in diverse environments [6].

### Prior Work on the Value and Benefits of D&I

Valuing D&I in the workforce goes beyond the basic requirements of skills and capabilities. Prior research suggests seven categories of diverse attitudes and perceptions: (1) diversity sensitivity, (2) integrity with a difference, (3) interaction variations, (4) valuing differences, (5) team inclusion, (6) managing conflict over differences, and (7) embedding inclusion [20]. Diversity focuses on the makeup of a population or its demographics, while inclusion encompasses involvement, engagement, and integration into organizational processes [21]. It is vital to create a supportive environment that is diverse, respectful, and inclusive [20]. Such an environment eases the expression of dissenting opinions, is open to new problem-solving approaches, encourages innovative thinking, and more effectively avoids the dangers of groupthink, thereby opening doors for innovation and creativity-based organizational culture and business performance [22]. Diverse customers are often more loyal to diverse workforces and businesses [11]. Thus, through diversity, companies create organizational capabilities beyond their collective talents and skills, and can be more responsive to a comprehensive system of values and customers in a competitive marketplace [23]. To illustrate, a diverse and inclusive organization can potentially tap into the disposable income of African Americans in the United States, which reached US $1.2 trillion in 2018 [24], and the buying power of Asian Americans, which topped US $1 trillion in 2018 [25].

Although diversity has attracted substantial research attention, significant barriers and difficulties often accompany its implementation [26]. A workable approach begins with embedding inclusiveness in all aspects of an organization’s culture, starting with recruiting different races, genders, sexual orientations, national origins, and religions. It also requires a conscious shift toward a culture in which policies and procedures provide opportunities for all employees to excel [27].

Diversity goes beyond the traditional “black and white” [28]. In addition to addressing observable attributes of inclusiveness such as race, invisible attributes such as religion, values, and beliefs are also important features of organizational culture to promote inclusiveness actively [29]. For instance, gender differences in the professional workforce have decreased considerably. Women now represent 47% of the US workforce and 52% of all managerial and professional positions [30]. Technology-driven, gender-fair hiring processes in many organizations have contributed to this trend [31]. In addition to hiring more women to improve diversity, there is an increasing trend of better representation among racial and ethnic minorities, immigrants, and people with disabilities in the US labor market. A 2018 study by Accenture found that the US economy could grow up to US $25 billion if more people with disabilities were to join the labor force [32]. US regulations also require federal contractors to hire more workers with disabilities to avoid penalties [33]. There is a myth that hiring people with disabilities will cost more, which is a concern among organizations with low revenue levels. However, research has shown that more than 30% of the accommodations for workers with disabilities do not require additional expenditures, even after purchasing assistive technologies [34,35]. Nevertheless, valuing D&I must move beyond the surface or visible attributes to encompass different cultural and situational values and behaviors [36]. Ultimately, such efforts must become embedded within the organizations to be successful.

Firms outside of health care (eg, Apple, Google, IBM) recognize the benefits of diversity [37]. Research has shown that a discriminatory work environment can hinder an organization’s ability to build and equip the workforce it needs, leading to decreased productivity and performance. Conversely, proactively valuing D&I can attract the best talent and create an environment of belongingness and respect [36].

Health care workforce diversity needs to improve to successfully treat a greater variety of patients, from increasing care reach to improved satisfaction for racial and ethnic minority patients. Accessibility to underserved patients through a diverse workforce will bring health care closer to African American, Hispanic, and Native American communities [38]. Patients treated by physicians of their own racial or ethnic background are more likely to report receiving higher-quality care [39]. Improving access, care, quality, and reach all have significant implications for the long-term success of the health care sector in the United States.

### Pathways to Equip the Workforce: Improve, Recruit, and Collaborate

What is the starting point toward greater workforce diversity? Undoubtedly, schools and universities are the formative platforms to inculcate D&I in young minds through examples, demonstrations, and practices [6]. Diverse classrooms broaden perspectives, promote active thinking, foster intellectual engagement, develop social skills, teach empathy, and improve racial understanding, all of which are essential for embracing diversity [40]. At the same time, organizations need to put more significant pressure on the education system to drive diversity. We consider three pathways to achieve this.

First, existing employees must acquire the necessary skill sets and diversity training. Programs such as “returnships,” in which experienced professionals take career breaks for training through professional and executive development programs, can help to promote and equip a more diverse workforce [41].

Second, technologies have made the recruitment process more efficient. Platforms such as LinkedIn and other social media avenues have become instrumental in finding talent. While health systems rely on traditional recruitment processes, using emerging channels to discover new talent could prove helpful.

Third, reaching out to and collaborating with universities can effectively expand the talent pool to recruit. This may start at the beginning of an education cycle, continue through projects and internships, and result in hiring from the collection of students engaged with the organization through these avenues.

For large health systems with diverse customers, a diverse base of employees is required. The revenue status of a health system can change its recognition of the direct link between diversity and performance. Major teaching health systems, as knowledge-based organizations, may have more proactive organizational cultures and reputations for openness, which will help them attract talent regardless of nationality or ethnic background. Macro factors such as increased mobility due to climate change and changing economic situations portend more women, more people of color, and more immigrant workers in the United States over the next 25 years [42]. To broaden recruitment to reflect the composition of society and the spread of business operations, organizations will need more women and people from different ethnic origins. In this context, understanding what health systems are doing to diversify their workforces remains an open question. In addition, due to social distancing policies implemented during the COVID-19 pandemic, digital transformations such as virtual teams and telehealth pose new challenges for collaboration. Diverse backgrounds among virtual collaborators, if managed well, can promote better learning to achieve more efficient outcomes [43]. Recognizing this potential will enhance remote working both during and after the COVID-19 pandemic.

The question remains as to which one or more of the three pathways mentioned above—improve, recruit, and collaborate—can effectively meet the challenges of D&I requirements. Identifying and assessing effective pathways will help instill appropriate plans in health systems. For example, explicitly valuing D&I will motivate organizations to develop long-term career plans to retain talent [44]. Organizations can better equip existing employees by developing internal training and education programs [20]. The critical element is an individual’s openness to change, which can be improved through training [45]. At the same time, it is also essential to recruit new employees, as having a diversity of work experience is a helpful way to refresh organizational culture. Finally, external collaboration with strategic partners benefits allying partners’ resources, including human resources [46]. This study further explores these three pathways to equip the workforce better—improving, recruiting, and collaborating—and their relative associations with business and service-oriented benefits.

The pathway model has been used in previous studies on diversity [47]. A common framework is diversity practices–pathways–performance [48]. Following this framework, we considered business and service benefits as the performance component. We examined the associations with three pathways: improving, recruiting, and collaborating. The two types of diversity practices are D&I strategy and talent strategy.

## Methods

### Data Collection

The effort to study the talent strategy in health systems is part of a broad project undertaken by the Health Administration Research Consortium at the Business School of the University of Colorado Denver. The idea of monitoring health systems emerged from observations and conversations with several chief executives of health systems during the COVID-19 pandemic. This research is part of the Health Systems’ Climate Study of 2021 conducted by the Health Administration Research Consortium [49]. The Climate Study aims to understand the current state of health systems in the United States following the COVID-19 pandemic. The objective was to collect and disseminate the insights of health systems’ CEOs to help inform policymakers, practitioners, and academic stakeholders as they collaborate to create ongoing strategies to help the industry respond to this pandemic and prepare for the next crisis.

A questionnaire was developed in December 2020 to collect data from health systems. We drew the survey items from prior literature, and questions were reworded to fit the health systems context. We sought input from knowledgeable researchers, consultants, and executives with the requisite expertise to design and evaluate the questions. The survey was pilot-tested, revised, and finalized in January 2021 with five top executives who are part of the Health Administration Program Advisory Board.

A contact list of CEOs was compiled from 624 health systems across the United States using multiple sources, contacts, professional connections, websites, and annual reports. The survey instrument was administered using a professional online survey platform, and was mapped to emails to the platform to create unique, trackable links for each health system. Email and phone solicitations were made in multiple rounds between January 25 and March 2, 2021. In addition, the authors called several CEOs and asked them to complete the survey instrument either online or in paper format. The researchers also requested CEOs who had participated in the survey to share the link with other CEO colleagues. A total of 148 responses were received, with a 24% response rate; however, 13 incomplete responses could not be used, leaving 135 usable responses. We address potential nonresponse bias in a later section.

The 135 health systems represented in this survey varied from 1 to 18 hospitals and from 176 to 75,000 employees. The annual revenue of the health systems in 2020 ranged from US $0.7 million to US $14 billion. The health systems represented US $300 billion in revenue and 1.1 million employees across the United States.

We then matched the survey data set with secondary data collected from the Agency for Healthcare Research and Quality compendium to construct a complete picture of the health systems. Our final sample included data from 124 health systems across the United States. We analyzed this combined data set, which yielded several important insights.

### Variables and Measures

Table 1 describes the variables used in this study. The two constructs of health systems’ workforce strategy focus are D&I STRATEGY and TALENT STRATEGY. The two constructs of health systems’ benefits are BUSINESS BENEFIT and SERVICE BENEFIT. These variables were each measured using 7-point Likert scales. We also tested the internal-consistency reliability of these multi-item variables using Cronbach α. The four α values were close to or greater than the recommended acceptable threshold of .70 for exploratory research [50].

The three variables used to measure the pathways to equip the workforce by health systems were IMPROVE (ie, improve current talent), RECRUIT (ie, recruit new talent), and COLLABORATE (ie, collaborate with universities). This study’s other independent and control variables represented several categories: size, region, teaching status, revenue, and several other system characteristics. We coded these variables (see Table 1) to reflect the attributes of a health system.

**Table 1:** Description of variables, including survey questions and coding scheme.

Variable			Description and coding		
**Question: “To what extent do the following dimensions describe how you address or plan to address in your company’s workforce strategy?”^a^**					
	D&I^b^ STRATEGY				Inclusion of diversity-relevant dimensions in your organization’s workforce strategy to attract talent: gender, ethnicity and race, disability, experience (Cronbach α=.60)
	TALENT STRATEGY				Inclusion of diversity-relevant dimensions in your organization’s workforce strategy: knowledge, attitude toward career and progression, personal quality or mindset, and adaptability (Cronbach α=.67)
**Question: “What benefits, if any, have your organization obtained from its strategy to promote diversity and inclusiveness?”^a^**					
	BUSINESS BENEFIT				Obtaining business benefits from promoting diversity and inclusiveness: enhancing business performance, strengthening brand reputation, and innovating internally or externally (Cronbach α=.70)
	SERVICE BENEFIT				Obtaining service benefits from promoting diversity and inclusiveness: enhance customer satisfaction, serve customer needs, leverage technology advancements for services, and compete in new industries (Cronbach α=.83)
**Three pathways to equip the health systems workforce: “How will you address talent needs in your organization?” ^a^**					
	IMPROVE				Returnships of existing employees to acquire new skills
	RECRUIT				Use multiple channels to find and recruit workers (ie, aspirations to discover new talent for health systems through emerging digital channels and traditional recruitment channels)
	COLLABORATE				Reach out and collaborate with universities to find new talent and establish plans to train students
**Coding of contingent variables**					
	**SIZE**				The size variable is measured using the total beds managed by the health system across all hospitals, reported by AHRQ^c^ Hospital Compendium
		SIZE_B-SMALL		Health system has fewer than 100 beds	
		SIZE_B-MEDIUM,		Health system has 100 to 400 beds	
		SIZE_B-LARGE		Health system has more than 400 beds	
	**REGION**				Following the Census Bureau’s categorization, the region variable is coded based on the health system’s primary location in the United States
		REGION-NE		Health system in the Northeast	
		REGION-MW		Health system in the Midwest	
		REGION-SOUTH		Health system in the South	
		REGION-WEST		Health system in the West	
	**TEACHING**				The teaching variable is coded based on the teaching status of a health system
		TEACHING-NON		Nonteaching health system	
		TEACHING-MINOR		Minor teaching health system	
		TEACHING-MAJOR		Central teaching health system	
	**REVENUE**				The revenue variable of the health system is measured using its annual revenue across all hospitals
		REVENUE-LOW		Revenue less than US $2 billion	
		REVENUE-MEDIUM		Revenue US $2-5 billion	
		REVENUE-HIGH		Revenue more than US $5 billion	
	HIGH-DSH-HOSP				The health system includes at least one high-discharge-patient-percentage hospital: 1=yes, 0=no
	HIGH-BURDEN-SYS				Health system-wide uncompensated care burden flag: 1=yes, 0=no
	HIGH-BURDEN-HOSP				The health system includes at least one high uncompensated care burden hospital: 1=yes, 0=no
	OWNERSHIP				Predominantly investor-owned hospitals: 1=yes, 0=no
	PHYSICIANS				The number of physicians in the health system is measured by the number of physicians reported by the AHRQ Hospital Compendium
	HOSPITALS				This variable is measured by the number of hospitals the health system has reported by the AHRQ Hospital Compendium

^a^Responses reflect a 7-point Likert scale from 1=strongly disagree to 7=strongly agree.

^b^D&I: diversity and inclusion.

^c^AHRQ: Agency for Healthcare Research and Quality.

The size variable measures the number of beds in a given health system (SIZE_B-SMALL, SIZE_B-MEDIUM, SIZE_B-LARGE). The region variable reflects the location of a health system (REGION-NE, REGION-MW, REGION-SOUTH, REGION-WEST). The teaching status variable assesses how a health system operates in association with a teaching program (TEACHING-NON, TEACHING-MINOR, TEACHING-MAJOR). The revenue variable measures the annual revenue of a health system (REVENUE-LOW, REVENUE-MEDIUM, REVENUE-HIGH). Finally, we included variables to capture the high discharge levels of the health systems (HIGH-DSH-HOSP), uncompensated care burden (HIGH-BURDEN-SYS and HIGH-BURDEN-HOSP), ownership status (OWNERSHIP), number of physicians (PHYSICIANS), and number of hospitals (HOSPITALS). Table 1 presents complete information about the variables in our study.

### Sample Statistics

The descriptive statistics and pairwise correlations among the key variables used in this study are shown in Table 2 and Table 3, respectively. As shown in Table 2, health systems, on average, value a talent strategy for improving employees’ skills and capabilities more than a D&I strategy. The most popular pathway to equip a workforce is through collaboration with universities, followed by recruitment, and then by improving the current workforce.

In addition, to ensure there was no nonresponse bias, we compared the characteristics of responding and nonresponding health systems. As shown in Table 4, the *t* test results for all comparisons indicated no significant difference between respondents and nonrespondents.

**Table 2:** Summary statistics of the variables (N=124).

Variables^a^	Mean (SD)	Range
D&I^b^ STRATEGY	4.62 (9.4)	2.3-6.5
TALENT STRATEGY	4.87 (1.10)	2.2-6.5
BUSINESS BENEFIT	5.35 (0.94)	1.7-7.0
SERVICE BENEFIT	4.67 (1.28)	2.0-6.5
IMPROVE	4.49 (1.35)	1-7
RECRUIT	4.67 (1.51)	1-7
COLLABORATE	4.82 (1.36)	2-7
SIZE_B-SMALL	0.09 (0.28)	0-1
SIZE_B-MEDIUM	0.37 (0.49)	0-1
SIZE_B-LARGE	0.54 (0.50)	0-1
REGION-NE	0.22 (0.42)	0-1
REGION-MW	0.24 (0.43)	0-1
REGION-SOUTH	0.35 (0.48)	0-1
REGION-WEST	0.18 (0.38)	0-1
TEACHING-NON	0.30 (0.46)	0-1
TEACHING-MINOR	0.48 (0.50)	0-1
TEACHING-MAJOR	0.22 (0.41)	0-1
REVENUE-LOW	0.61 (0.49)	0-1
REVENUE-MEDIUM	0.23 (0.43)	0-1
REVENUE-HIGH	0.15 (0.35)	0-1
HIGH-DSH-HOSP	0.33 (0.47)	0-1
HIGH-BURDEN-SYS	0.20 (0.40)	0-1
HIGH-BURDEN-HOSP	0.30 (0.46)	0-1
OWNERSHIP	0.02 (0.13)	0-1
PHYSICIANS	1.84 (0.80)	1-3
HOSPITALS	1.50 (0.77)	1-3

^a^See Table 1 for variable descriptions.

^b^D&I: diversity and inclusion.

**Table 3:** Pairwise correlations among key variables (N=124).

Variables^a^	1	2	3	4	5	6	7	8	9	10	11	12	13	14	15	16	17
1. D&I STRATEGY	1.00	–0.04	–0.07	*0.31* ^b^	*0.41*	*0.80*	*0.84*	0.11	0.11	–0.01	0.01	0.11	0.08	–0.08	0.03	0.11	0.14
2. TALENT STRAT.	–0.04	1.00	*0.64*	*0.52*	*0.29*	0.04	–*0.29*	–0.07	–*0.19*	0.002	–0.13	–0.10	–0.04	0.04	–0.12	–0.04	–0.02
3. BUSINESS BENEF.	0.07	*0.64*	1.00	*0.79*	0.11	*0.22*	–*0.16*	–0.08	–*0.17*	–0.001	–0.15	–0.01	–0.12	0.11	–0.02	0.001	–0.04
4. SERVICE BENEF.	*0.31*	*0.52*	*0.79*	1.00	*0.27*	*0.46*	0.06	–0.09	–0.11	0.05	–*0.18*	0.01	–0.02	0.11	–0.05	–0.02	0.01
5. IMPROVE	*0.41*	*0.29*	0.11	*0.27*	1.00	*0.15*	*0.16*	0.04	0.12	0.10	–0.09	–0.03	0.002	0.14	–0.10	–0.03	–0.05
6. RECRUIT	*0.80*	0.04	*0.22*	*0.46*	*0.15*	1.00	*0.58*	0.03	0.06	0.07	–0.10	0.02	0.09	–0.14	0.02	0.03	0.15
7. COLLABORATE	*0.84*	–*0.29*	–*0.16*	0.06	*0.16*	*0.58*	1.00	0.12	*0.15*	–0.08	0.07	0.11	0.01	–0.11	0.02	0.12	0.13
8. SIZE	0.11	–0.07	–0.08	–0.09	0.04	0.03	0.12	1.00	0.07	–*0.19*	*0.53*	*0.53*	*0.19*	0.09	*0.28*	*0.70*	*0.49*
9. REGION	0.11	–*0.19*	–*0.17*	–0.11	0.12	0.06	*0.15*	0.07	1.00	0.07	–0.06	0.003	*0.16*	*0.22*	*0.23*	–0.04	0.03
10. OWNERSHIP	–0.01	0.002	–0.001	0.05	0.10	0.07	–0.08	–*0.19*	0.07	1.00	–0.07	–0.09	0.05	–0.06	–0.08	–0.05	–0.08
11. TEACHING	0.01	–0.13	–0.15	–*0.18*	–0.09	–0.10	0.07	*0.53*	–0.06	-.07	1.00	*0.34*	*0.42*	–0.05	*0.20*	*0.57*	*0.26*
12. REVENUE	0.11	–0.10	–0.01	0.01	–0.03	0.02	0.11	*0.53*	0.003	-.09	*0.34*	1.00	0.08	–0.05	0.07	*0.62*	*0.41*
13. HIGH-DSH-HOSP.	0.08	–0.04	–0.12	–0.02	0.002	0.09	0.01	*0.19*	*0.16*	.05	*0.42*	0.08	1.00	–0.01	*0.19*	*0.23*	*0.18*
14. HIGH-BURD.-SYS	–0.08	0.04	0.11	0.11	0.14	–0.14	–0.11	0.09	*0.22*	-.06	–0.05	–0.05	–0.01	1.00	*0.42*	–0.10	*0.20*
15. HIGH-BURD.-HOSP	0.03	–0.12	–0.02	–0.05	–0.10	0.02	0.02	*0.28*	*0.23*	-.08	*0.20*	0.07	*0.19*	*0.42*	1.00	*0.18*	*0.31*
16. PHYSICIANS	0.11	–0.04	0.001	–0.02	–0.03	0.03	0.12	*0.70*	–0.04	-.05	*0.57*	*0.62*	*0.23*	–0.10	*0.18*	1.00	*0.57*
17. HOSPITALS	0.14	–0.02	–0.04	0.01	–0.05	0.15	0.13	*0.49*	0.03	-.08	*0.26*	*0.41*	*0.18*	–*0.20*	*0.31*	*0.57*	1.00

^a^See Table 1 for variable descriptions.

^b^Values in italics indicate a significant correlation at *P*<.10.

**Table 4:** Characteristics of responding and nonresponding health systems.

Characteristics^a^			Respondents (n=124), n (%)	Nonrespondents (n=511), n (%)	*t* value
**Size**					
	Small (6-99 beds)	11 (8.8)		41 (8.0)	–0.19
	Medium (100-399 beds)	46 (37.1)		210 (41.1)	–0.56
	Large (≥400 beds)	67 (54.0)		260 (50.1)	1.41
**Region**					
	Northeast	27 (21.8)		118 (23.1)	0.07
	Midwest	30 (24.2)		132 (25.8)	0.55
	South	45 (36.3)		169 (33.1)	–0.48
	West	22 (17.7)		92 (18.0)	–0.12
**Physicians**					
	Small (51-199 physicians)	50 (40.3)		189 (37.0)	–0.74
	Medium (200-999 physicians)	41 (33.1)		204 (40.0)	–0.69
	Large (≥1000 physicians)	33 (26.7)		118 (23.1)	1.53
**Hospitals**					
	Small (1-3 hospitals)	83 (66.9)		338 (66.1)	–1.27
	Medium (4-6 hospitals)	20 (16.1)		66 (12.9)	–0.02
	Large (≥7 hospitals)	21 (16.9)		107 (20.9)	0.81
**Ownership status**					
	Investor-owned	3 (2.4)		15 (2.9)	–0.85
	Noninvestor-owned	121 (97.6)		496 (97.1)	0.85
**Teaching status**					
	Major teaching	29 (23.4)		138 (27.0)	–0.15
	Minor teaching	58 (46.8)		225 (44.0)	–0.61
	Nonteaching	37 (29.8)		148 (29.0)	0.85

^a^The numbers of physicians and hospitals are presented in this table in different categories for easy comparison across respondents and nonrespondents.

### Statistical Analysis

We used ordered logit regressions to estimate (1) the relationship between specific hospital characteristics and workforce-strategy focus as well as diversity benefits, (2) the relationship between workforce-strategy focus and pathways to equip the workforce, and (3) the mediating effects of workforce choices on the relationship between workforce strategy focus and diversity-driven business and service outcomes. We used ordered logit regressions because the dependent variables are ordinal. This approach does not assume equal intervals between levels of the dependent variable. The ordered logit model is as follows:

Y_i_^*^=*βX_i_*+*e_i_*,

where Y_i_^*^ is the propensity of respondents to indicate higher levels of the dependent variables, *X_i_* is a set of explanatory variables, β a vector of parameters, and *e_i_* are disturbances (errors).

We do not observe Y_i_*; instead, we observe the ordinal dependent variable *Y_i_*. Depending on the values of thresholds or cut-off points *τ_m_*_–1_ and *τ_m_*, the probability distribution of *Y_i_* is as follows:

Pr(*Y_i_*=*m*|**X**_i_=*F*(*τ_m_*–X*β*)–*F*(*τ_m_*_–1_–X*β*)

### Ethical Considerations

An ethics review was not applicable for this study. The data used was received through a leading professional consulting firm that anonymizes and provides secondary firm-level data for research and analysis to draw insights.

## Results

### Estimation Outcomes

The first two columns in Table 5 display the results from the ordered logit-model estimations that describe the relationship between contingent factors and health systems’ workforce strategy focus. The remaining two columns in Table 5 present the results on health systems’ diversity-enabled benefits.

First, the results indicate that compared to small-sized health systems, medium-sized health systems are less likely to value diversity and inclusiveness in their D&I strategies (*P*<.001). Conversely, large-sized health systems are more likely to value D&I strategies than small-sized health systems (*P*=.002). There are some differences between health systems located in the Northeast and West, insofar as those in the West tend to focus more on diversity and inclusiveness (*P*=.001).

Second, when the health system includes at least one high-discharge-patient-percentage hospital, it tends to value D&I more (*P*<.001). The results also showed that high-revenue health systems seem to value D&I less than low-revenue health systems. In addition, health systems with a system-wide high uncompensated care burden tend to value D&I less.

These results differ from the estimation results of the contingent factors on valuing a talent-acquisition strategy (Table 5). In terms of a workforce strategy focus, there seem to be no differences in health systems concerning size, ownership status, discharge, uncompensated care burden, and the number of physicians and hospitals. Region and revenue level yielded the most significant differences. The results indicate that health systems in the Northeast emphasize employees’ skills and capabilities more than those located in the South and West. In addition, compared to low-revenue health systems, medium- and high-revenue health systems tend to place less emphasize on a talent-acquisition strategy (*P*<.001).

The last columns in Table 5 show the associations between health system characteristics and business and service benefits (while valuing D&I). The results of size and revenue were consistent for both types of benefits. For both business benefits (*P*<.001) and service benefits (*P*<.01), small-sized health systems tend to gain compared with medium- and large-sized health systems. Further, high-revenue health systems are more likely to gain both types of benefits than low-revenue systems (*P*<.001).

We also found some differences between these two benefits across health systems. For the business, investor-owned health systems, health systems with medium revenue (vs low revenue), health systems with at least one high-discharge-patient-​percentage hospital, and health systems with a system-wide uncompensated care burden tend to gain more benefits, whereas health systems with more hospitals are more likely to gain fewer business development benefits due to a diversity strategy. For service-oriented benefits, some differences were found according to region. Compared with health systems located in the Northeast, those in the South and in the West seem to gain fewer service-improvement benefits (Table 5).

Table 6 shows the different relationships between the three workforce pathways and the D&I and talent strategies. The results indicate a significant and negative relationship between D&I STRATEGY and COLLABORATE, but a significant and positive relationship between TALENT STRATEGY and RECRUIT. The relationship between TALENT STRATEGY and COLLABORATE was significant and positive. The relationships between the two strategies and the IMPROVE pathway as well as the relationship between D&I STRATEGY and the RECRUIT pathway were not significant.

Table 7 displays the mediating effects of the three workforce pathways (ie, IMPROVE, RECRUIT, and COLLABORATE) on the direct relationship between D&I and talent strategies and the business benefit. Analysis of the mediating models using Sobel Goodman tests, which determine whether a variable carries (or mediates) the effect of an independent variable to the dependent variable (the outcome of interest), showed that overall, IMPROVE has a higher mediating effect (44%) than COLLABORATE (4%) and RECRUIT (7%) between a D&I strategy and business benefit. Similarly, IMPROVE has a higher mediating effect (13%) than COLLABORATE (5%) and RECRUIT (1%) between a talent strategy and business benefit.

Table 8 shows the mediating effects of the three workforce pathways (ie, IMPROVE, RECRUIT, and COLLABORATE) on the direct relationship between D&I and talent strategies on service benefit. Analysis of the mediating models using Sobel Goodman tests showed that overall, IMPROVE has a higher mediating effect (27%) than COLLABORATE (2%) and RECRUIT (0.05%) between a D&I strategy and service benefit. Similarly, IMPROVE has a higher mediating effect (26%) than COLLABORATE (0.06%) and RECRUIT (0.02%) between a talent strategy and service benefit.

**Table 5:** Differences across health systems^a^.

Variables^b^	D&I^c^ strategy^d^		Talent strategy^e^			Business benefit^f^		Service benefit^g^	
	Coefficient (SE)	*P* value	Coefficient (SE)	*P* value	Coefficient (SE)		*P* value	Coefficient (SE)	*P* value
SIZE_B-MEDIUM	–0.685 (0.074)	<.001	–0.204 (0.516)	.69	–1.035 (0.167)		<.001	–1.329 (0.447)	.003
SIZE_B-LARGE	0.342 (0.110)	.002	0.411 (0.957)	.67	–0.377 (.057)		<.001	–1.441 (0.472)	.002
REGION-MW	0.069 (0.304)	.82	–0.481 (0.572)	.40	–0.772 (0.948)		.42	–0.945 (0.750)	.21
REGION-SOUTH	0.180 (0.433)	.68	–1.363 (0.403)	.001	–0.698 (0.622)		.26	–1.597 (0.496)	.001
REGION-WEST	0.482 (0.144)	.001	–0.761 (0.106)	<.001	–0.009 (0.756)		.99	–1.224 (0.558)	.03
TEACHING-MINOR	–0.228 (0.241)	.34	–0.016 (0.419)	.97	0.207 (0.744)		.78	–0.393 (1.039)	.71
TEACHING-MAJOR	–0.743 (1.155)	.52	–0.727 (0.394)	.07	–0.673 (0.565)		.23	–1.304 (0.816)	.11
REVENUE-MEDIUM	0.622 (0.912)	.50	–0.784 (0.042)	<.001	0.339 (0.122)		.005	–0.169 (0.130)	.19
REVENUE-HIGH	–0.241 (0.104)	.02	–0.338 (0.047)	<.001	0.662 (0.098)		<.001	0.188 (0.046)	<.001
HIGH-DSH-HOSP	0.359 (0.061)	<.001	0.298 (0.364)	.41	0.424 (0.187)		.02	0.038 (0.508)	.94
HIGH-BURDEN-SYS	–0.552 (0.250)	.03	0.463 (0.679)	.50	0.675 (0.127)		<.001	0.780 (0.526)	.14
HIGH-BURDEN-HOSP	–0.100 (0.454)	.83	–0.482 (0.708)	.50	–0.302 (0.456)		.51	0.102 (0.264)	.70
OWNERSHIP	–0.258 (0.290)	.37	0.504 (3.485)	.89	1.559 (0.655)		.02	–0.397 (3.235)	.90
PHYSICIANS	–0.092 (0.355)	.80	–0.074 (0.307)	.81	–0.102 (0.344)		.77	0.267 (0.218)	.22
HOSPITALS	0.031 (0.124)	.80	0.189 (0.251)	.45	–0.248 (0.112)		.03	0.173 (0.164)	.29

^a^The results of the cut points are omitted for brevity.

^b^See Table 1 for variable descriptions.

^c^D&I: diversity and inclusion.

^d^Pseudo *R*^2^=0.0247 (n=124 observations).

^e^Pseudo *R*^2^=0.0298 (n=124 observations).

^f^Pseudo *R*^2^=0.0282 (n=124 observations).

^g^Pseudo *R*^2^=0.0401 (n=123 observations).

**Table 6:** Workforce strategy focus and workforce pathways^a^.

Variables^b^	IMPROVE pathway^c^		RECRUIT pathway^d^			COLLABORATE pathway^e^	
	Coefficient (SE)	*P* value	Coefficient (SE)	*P* value	Coefficient (SE)		*P* value
D&I^f^ STRATEGY	–0.059 (0.394)	.88	–0.098 (0.153)	.52	–0.134 (0.036)		<.001
TALENT STRATEGY	–0.099 (0.110)	.37	0.950 (0.156)	<.001	0.523 (0.259)		.04
SIZE	0.169 (0.195)	.37	–0.356 (0.512)	.49	–0.954 (0.571)		.10
REGION	0.108 (0.248)	.66	0.121 (0.136)	.37	–0.315 (0.096)		.001
OWNERSHIP	1.727 (1.080)	.11	1.071 (0.351)	.002	0.018 (1.378)		.99
TEACHING	–0.256 (0.086)	.003	–0.240 (0.165)	.15	–0.364 (0.153)		.02
REVENUE	–0.087 (0.226)	.70	0.025 (0.107)	.81	0.704 (0.219)		.001
HIGH-DSH-HOSP	0.330 (0.108)	.002	0.133 (0.300)	.66	0.132 (0.286)		.64
HIGH-BURDEN-SYS	0.852 (0.267)	.001	0.193 (0.165)	.24	–0.275 (0.320)		.39
HIGH-BURDEN-HOSP	–0.847 (0.517)	.10	–0.483 (0.269)	.07	1.270 (0.582)		.03
PHYSICIANS	–0.202 (0.054)	<.001	–0.033 (0.155)	.83	0.431 (0.487)		.38
HOSPITALS	0.113 (0.109)	.30	0.351 (0.160)	.03	0.027 (0.188)		.88

^a^The results of the cut points are omitted for parsimony.

^b^See Table 1 for variable descriptions.

^c^Pseudo *R*^2^=0.0336 (n=124 observations).

^d^Pseudo *R*^2^=0.0940 (n=124 observations).

^e^Pseudo *R*^2^=0.0856 (n=124 observations).

^f^D&I: diversity and inclusion.

**Table 7:** Associations of workforce pathways and business benefits^a^.

Variables^b^	Model 1^c^		Model 2^d^			Model 3^e^			Model 4^f^			Model 5^g^	
	Coefficient (SE)	*P* value	Coefficient (SE)	*P* value	Coefficient (SE)		*P* value	Coefficient (SE)		*P* value	Coefficient (SE)		*P* value
D&I^h^ STRATEGY	0.496 (0.335)	.14	0.604 (0.264)	.02	0.505 (0.263)		.06	0.529 (0.346)		.13	0.624 (0.270)		.02
TALENT STRATEGY	1.331 (0.252)	<.001	1.500 (0.268)	<.001	1.093 (0.248)		<.001	1.274 (0.242)		<.001	1.334 (0.300)		<.001
IMPROVE	—^i^	—	0.766 (0.171)	<.001	—		—	—		—	0.597 (0.218)		.006
RECRUIT	—	—	—	—	0.416 (0.012)		<.001	—		—	0.187 (0.010)		<.001
COLLABORATE	—	—	—	—	—		—	0.444 (0.076)		<.001	0.282 (0.108)		.009
SIZE	–0.292 (0.115)	.01	–0.386 (0.213)	.07	–0.238 (0.135)		.08	–0.104 (0.194)		.59	–0.248 (0.258)		.34
REGION	–0.118 (0.100)	.24	–0.234 (0.051)	<.001	–0.169 (0.082)		.04	–0.048 (0.083)		.56	–0.174 (0.073)		.02
OWNERSHIP	0.252 (0.663)	.70	–0.326 (1.184)	.78	–0.041 (0.832)		.96	0.348 (0.540)		.52	–0.294 (1.065)		.78
TEACHING	–0.248 (0.573)	.67	–0.156 (0.553)	.78	–0.177 (0.608)		.77	–0.170 (0.595)		.78	–0.120 (0.610)		.85
REVENUE	0.152 (0.067)	.02	0.239 (0.018)	<.001	0.181 (0.079)		.02	0.056 (0.170)		.74	0.185 (0.107)		.08
HIGH-DSH-HOSP	–0.389 (0.536)	.47	–0.437 (0.523)	.40	–0.416 (0.454)		.36	–0.357 (0.487)		.46	–0.387 (0.469)		.41
HIGH-BURDEN-SYS	0.567 (0.107)	<.001	0.429 (0.138)	.002	0.546 (0.136)		<.001	0.669 (0.176)		<.001	0.541 (0.105)		<.001
HIGH-BURDEN-HOSP	0.218 (0.390)	.58	0.428 (0.248)	.09	0.328 (0.296)		.27	–0.111 (0.574)		.85	0.197 (0.285)		.49
PHYSICIANS	0.413 (0.560)	.46	0.388 (0.609)	.52	0.419 (0.545)		.44	0.253 (0.712)		.72	0.327 (0.677)		.63
HOSPITALS	–0.303 (0.205)	.14	–0.182 (0.255)	.48	–0.385 (0.173)		.03	–0.248 (0.257)		.34	–0.179 (0.273)		.51

^a^The results of the cut points are omitted for brevity.

^b^See Table 1 for variable descriptions.

^c^Pseudo *R*^2^=0.1209 (n=124 observations).

^d^Pseudo *R*^2^=0.1539 (n=124 observations).

^e^Pseudo *R*^2^=0.1391 (n=124 observations).

^f^Pseudo *R*^2^=0.1334 (n=124 observations).

^g^Pseudo *R*^2^=0.1638 (n=124 observations).

^h^D&I: diversity and inclusion.

^i^Not included in model.

**Table 8:** Associations of workforce pathways and service benefits.a

Variables^b^	Model 1^c^		Model 2^d^					Model 3^e^					Model 4^f^					Model 5^g^			
	Coefficient (SE)	*P* value		Coefficient (SE)		*P* value			Coefficient (SE)		*P* value			Coefficient (SE)		*P* value			Coefficient (SE)		*P* value
D&I^h^ STRATEGY	0.758 (0.088)	<.001	0.770 (0.039)		<.001		0.830 (0.076)			<.001		0.774 (0.152)			<.001		0.873 (0.081)			<.001	
TALENT STRATEGY	1.165 (0.192)	<.001	1.256 (0.208)		<.001		0.783 (0.139)			<.001		1.098 (0.182)			<.001		0.886 (0.155)			<.001	
IMPROVE	—^i^	—	0.655 (0.059)		<.001		—			—		—			—		0.448 (0.024)			<.001	
RECRUIT	—	—	—		—		0.762 (0.080)			<.001		—			—		0.653 (0.100)			<.001	
COLLABORATE	—	—	—		—		—			—		0.434 (0.323)			.18		0.291 (0.357)			.42	
SIZE	–0.502 (0.350)	.15	–0.521 (0.364)		.15		–0.337 (0.165)			.04		–0.300 (0.501)			.55		–0.257 (0.378)			.50	
REGION	–0.204 (0.279)	.47	–0.337 (0.255)		.19		–0.330 (0.189)			.08		–0.132 (0.269)			.62		–0.351 (0.204)			.09	
OWNERSHIP	1.193 (0.507)	.02	0.747 (0.830)		.37		0.766 (0.771)			.32		1.287 (0.290)			<.001		0.653 (0.723)			.37	
TEACHING	–0.314 (0.471)	.51	–0.356 (0.494)		.47		–0.353 (0.662)			.59		–0.281 (0.495)			.57		–0.327 (0.624)			.60	
REVENUE	0.387 (0.236)	.10	0.408 (0.234)		.08		0.406 (0.237)			.09		0.226 (0.272)			.41		0.358 (0.301)			.23	
HIGH-DSH-HOSP	0.030 (0.444)	.95	0.095 (0.446)		.83		0.121 (0.377)			.75		0.119 (0.407)			.77		0.118 (0.387)			.76	
HIGH-BURDEN-SYS	1.239 (0.325)	<.001	1.039 (0.468)		.03		1.116 (0.284)			<.001		1.311 (0.186)			<.001		0.999 (0.241)			<.001	
HIGH-BURDEN-HOSP	–0.429 (0.367)	.24	–0.166 (0.285)		.56		–0.104 (0.182)			.57		–0.767 (0.748)			.31		–0.045 (0.421)			.92	
PHYSICIANS	0.073 (0.453)	.87	0.121 (0.541)		.82		0.178 (0.437)			.68		0.025 (0.682)			.97		0.133 (0.628)			.83	
HOSPITALS	0.221 (0.343)	.52	0.323 (0.344)		.35		–0.007 (0.244)			.98		0.257 (0.396)			.52		0.042 (0.271)			.88	

^a^The results of the cut points are omitted for brevity.

^b^See Table 1 for variable descriptions.

^c^Pseudo *R*^2^=0.123 (n=123 observations).

^d^Pseudo *R*^2^=0.153 (n=123 observations).

^e^Pseudo *R*^2^=0.178 (n=123 observations).

^f^Pseudo *R*^2^=0.135 (n=123 observations).

^g^Pseudo *R*^2^=0.194 (n=123 observations).

^h^D&I: diversity and inclusion.

^i^Not included in model.

## Discussion

### Implications of Findings

Getting diversity right in the health care workforce remains a challenge, regardless of the widespread realization that D&I is critically important in this sector. Health systems lag in proactive plans, results-driven strategies, and subsequent implementations. Without these, the concept of D&I will be but a fad without any tangible results for decades to come.

This study explored the differences in D&I strategies across different health system characteristics. The findings suggest that health systems with fewer beds, those located in the western United States, with low revenues, with at least one high-discharge hospital, and a relatively low system-wide uncompensated care burden tend to value D&I more and are more likely to have a D&I strategy in place. Plausibly, these systems are driven by a focused strategy, locational alignments, and a manageable suite of complexities to instill D&I plans. Some of these differ from a talent-acquisition approach, indicating that health systems treat these two diversity practices differently. Regarding the diversity benefits, it seems that small health systems with comparatively high revenue have been able to gain both business- and service-related benefits; however, in other aspects of the health systems, the benefits vary across categories.

The most important contribution of this study has been to compare and contrast the three workforce pathways and their associations with benefits. The findings suggest that health systems that value *only* a D&I strategy may *not* rely on collaboration with universities to equip their workforces. However, health systems that value a talent strategy will look externally to recruit new workers and seek collaboration with universities.

While examining the pathways through mediation analyses, we established that the IMPROVE pathway is more effective than the RECRUIT and COLLABORATE pathways in enabling the diversity strategy to prompt business or service benefits. Moreover, these pathway effects go hand-in-hand with a talent strategy, indicating that both talent and diversity strategies need to be aligned to achieve the best results for a health system.

### Limitations and Directions for Further Research

This study has some limitations that future studies may be able to address. For example, we did not focus on the effects of internal issues (eg, management, coordination) on diversity. Furthermore, the opportunities and barriers to diversity strategies should be studied in detail. Relating diversity to well-known aspects of health care delivery, such as cost, quality, and patient-experience outcomes, is also essential. We also need to note that the 22% response rate is not very high, although it represents the US health systems’ population. Increasing response rates and covering all health systems in a study will require significant resources, and we may perform such a study in the future.

### Conclusions

The challenges and uncertainties that COVID-19 presented to health systems in the United States have been unprecedented. The pandemic has propelled many issues to the forefront, including diversity. It is time for health systems to address the diversity issue, which has been a point of conversation for more than two or three decades. However, little progress has been made to date, and few proactive strategies are in place, leading to a nondiverse workforce in US health care.

This study demonstrates that D&I efforts have numerous positive business and service outcomes. Regarding the methods to address the talent shortage, it seems that health systems that value D&I are less likely to seek external collaborations. This may be because external collaboration is not an effective way to promote D&I inside the health systems. A notable point is the importance of professional and executive training programs, and further education for instilling a D&I mindset, strategy, and pathways in a health system. This improvement pathway is beneficial for outcomes; however, diversity and talent-acquisition efforts must be aligned with recruitment to yield multiple benefits for health systems. Following these findings, our recommendations will help health systems establish a more diverse health care workforce and improve outcomes for a diverse population.

## Abbreviations

CEO: chief executive officer
D&I: diversity and inclusion
